# Short-term surgical and long-term survival outcomes after laparoscopic distal gastrectomy with D_2_ lymphadenectomy for gastric cancer

**DOI:** 10.1186/1471-230X-14-41

**Published:** 2014-02-25

**Authors:** Ke Chen, Yi-Ping Mou, Xiao-Wu Xu, Jia-Qin Cai, Di Wu, Yu Pan, Ren-Chao Zhang

**Affiliations:** 1 Department of General Surgery, Sir Run Run Shaw Hospital, School of Medicine, Zhejiang University, 3 East Qingchun Road, Hangzhou 310016, Zhejiang Province, China

**Keywords:** Stomach neoplasms, Gastrectomy, Laparoscopy, Lymphadenectomy, Survival

## Abstract

**Background:**

Laparoscopic distal gastrectomy (LDG) for gastric cancer has gradually gained popularity. However, the long-term oncological outcomes of LDG have rarely been reported. This study aimed to investigate the survival outcomes of LDG, and evaluate the early surgical outcomes of laparoscopy-assisted distal gastrectomy (LADG) and totally laparoscopic distal gastrectomy (TLDG).

**Methods:**

Clinical outcomes of 240 consecutive patients with gastric cancer who underwent LDG at our institution between October 2004 and April 2013 were analyzed. Early surgical outcomes of LADG and TLDG were compared and operative experiences were evaluated.

**Results:**

Of the 240 patients, 93 underwent LADG and 147 underwent TLDG. There were 109 T1, 36 T2, 31 T3, and 64 T4a lesions. The median follow-up period was 31.5 months (range: 4–106 months). Tumor recurrence was observed in 40 patients and peritoneal recurrence was observed most commonly. The 5-year disease-free survival (DFS) and overall survival (OS) rates according to tumor stage were 90.3% and 93.1% in stage I, 72.7% and 67.6% in stage II, and 34.8% and 41.5% in stage III, respectively. No significant differences in early surgical outcomes were noted such as operation time, blood loss and postoperative recovery between LADG and TLDG (*P* >0.05).

**Conclusions:**

LDG for gastric cancer had acceptable long-term oncologic outcomes. The early surgical outcomes of the two commonly used LDG methods were similar.

## Background

Gastric cancer is one of the most common causes of cancer-related death worldwide [1]. Although, adjuvant chemotherapy improves the survival of these patients [2,3], radical gastrectomy with regional lymph node dissection remains the only potentially curative treatment available for gastric adenocarcinoma [4,5].

Since it was first reported in 1994 [6], laparoscopic distal gastrectomy (LDG) for gastric cancer has undergone rapid development and gained popularity in the past 20 years due to its well-known advantages, which include a less painful course of recovery, earlier recovery, better cosmesis, and improved short-term quality of life over open gastrectomy [7-10]. Although the minimally invasive effect of LDG is excellent, the therapeutic effects in adenocarcinoma still lack support from long-term follow-up studies. The most common version of LDG is laparoscopy-assisted distal gastrectomy (LADG) and totally laparoscopic distal gastrectomy (TLDG). In both of these techniques, perigastric lymphadenectomy is performed under laparoscopy. However, the former requires an epigastrium auxiliary incision for safe en bloc extraction of the specimen, and to finish reconstruction of the digestive tract. The latter is characterized by an intracorporeal anastomosis without auxiliary incision and it is considered “incisionless”, with the exception of the trocar wounds. Whether these two different anastomosis procedures affect the short-term outcome of this type of surgery remains controversial [11-13].

The purpose of this study was to evaluate the long-term oncologic outcomes of patients who underwent LDG, focusing on postoperative recurrence and survival rates. In addition, the short-term surgical variables and outcomes of LADG and TLDG were compared to evaluate the advantages and disadvantages of these procedures.

## Methods

### Patients

Between October 2004 and April 2013, 251 consecutive patients who underwent LDG with D_2_ lymphadenectomy for gastric cancer at Sir Run Run Shaw Hospital were included in this study. The exclusion criteria included: (1) invasion of adjacent structures; (2) palliative resection or no R0 resection; (3) distant metastases (e.g. peritoneal metastasis or peritoneal lavage cytology positive for carcinoma cells, hepatic metastasis); and (4) not confirmed pathologically as adenocarcinoma.

Blood tests, chest X-rays, enhanced computed tomography scans of the abdomen and pelvis, and gastric endoscopy were performed before surgery. This study protocol was approved by our hospital’s ethics committee and informed consent was signed by each patient prior to surgery.

### Surgical procedure

The patient was placed in the supine position under general anesthesia. The surgeon stood on the right side of the patient. One assistant stood on the right side of the patient and held the laparoscope, and another stood on the left side of the patient. Carbon dioxide pneumoperitoneum was instituted through a Veress needle and set at 15 mmHg. One initial 10-mm trocar was inserted for laparoscopy below the umbilicus and another four trocars (one of 12 mm, three of 5 mm) were inserted into the left upper flank, left flank, right upper flank, and right flank quadrants, respectively; a total of five trocars were inserted, and arranged in a V-shape [14,15]. D_2_ lymphadenectomy was performed according to the Japanese gastric cancer guidelines which included No. 7, 8, 9, 10, 11p, 11d, and 12a, and 14v in addition to D_1_ dissection. Anastomosis methods included Billroth I or Billroth II gastrojejunostomy. The detailed lymphadenectomy and reconstruction procedure were described in our previously published articles [16,17].

### Data collection and follow-up evaluation

Demographics and perioperative data were retrospectively collected from hospital records and analyzed. Clinical and pathological staging were determined according to the American Joint Committee on Cancer (7th edition), using the tumor-node-metastasis (TNM) classification scheme. Adjuvant chemotherapy with 5-fluorouracil (5-FU)-based regimens (mostly 5-FU with cisplatin) was recommended to all eligible patients, except those with stage I cancers. Follow-up data were collected for at least 3 years, including alternating semiannual abdominopelvic CT scans or ultrasound examinations. An endoscopic surveillance was performed annually or earlier if the patient had symptoms or there was any suspicion of recurrence. Recurrence patterns included peritoneal, locoregional, lymph node and hematogenous. Peritoneal recurrences included peritoneal seedlings or Krukenberg’s tumors. Locoregional recurrences included tumors in adjacent organs, remnant stomach or anastomoses. Hematogenous recurrences included tumors in other distant sites, such as liver, lung, bone, and brain.

### Statistical analysis

Quantitative data were expressed as the means ± standard deviations (SD). The differences in the measurement data were compared using the Student’s *t* test, and comparisons between groups were tested using the χ^2^ test or the Fisher exact probability test. Disease-free survival (DFS) and overall survival (OS) rates were calculated by the Kaplan–Meier method using SPSS software, version 18.0 (SPSS Inc, Chicago, United States). DFS was defined as the time from surgery to the time of recurrence of the original gastric cancer or development of a second malignancy. OS was defined as the time from surgery to date of death from any cause. *P* < 0.05 was considered statistically significant.

## Results

### Demographic and clinicopathologic characteristics

Among the 251 patients, 11 were excluded: five could not undergo R0 resection (three with tumor invasion of adjacent structures, two with conglomeration of lymph nodes), three had distant metastases, and three did not have adenocarcinoma (two neuroendocrine carcinomas, one lymphoma). After excluding these 11 patients, 240 with curative intent were included in this study.

Demographic and clinicopathologic characteristics are listed in Table 1. The mean age of the patients was 59.3 years (range, 30–81 years) and the male: female ratio was 2.5:1 (171 males). Mean body mass index (BMI) of the patients was 22.7 kg/m^2^ (range, 14.5–32.9 kg/m^2^). Slightly more than a third (83/240; 34.6%) of the patients had comorbidities, the most common being hypertension. Of these 240 patients, 93 underwent LADG and 147 underwent TLDG. From the pathologic results, 45.4% of patients had lesions that were staged as T1, 50.8% were staged as N0, and 53.3% had stage I neoplasms. Approximately 55% of patients had advanced gastric cancer, defined as tumor invasion into the proper muscular layer.

**Table 1:** Clinical characteristics and pathologic features

**Variables**	**Values**
Gender (male/female)	171/69
Age (years)	59.3 ± 10.7
BMI (kg/m^2^)	22.7 ± 3.0
ASA classification (I/II/III)	118/108/14
Comorbidities	83
Hypertension	52
Diabetes mellitus	19
Cardiovascular	16
Pulmonary	9
Liver	7
Others	8
Tumor size (cm)	3.6 ± 2.0
Histology (differentiated/undifferentiated)	131/109
T stage (T1/T2/T3/T4a)	109/36/31/64
N stage (N0/N1/N2/N3)	122/57/36/25
TNM stage (I/II/III/IV)	128/45/67/0

### Operative findings and postoperative clinical course

The operative findings and postoperative clinical course data are shown in Table 2. The mean operation time was 231.1 min (range, 150–380 min), with a mean blood loss of 136.3 mL (range, 20–420 mL). The mean number of retrieved lymph nodes per patient was 30.4 (range, 16–66). The mean proximal and distal resection margins were 4.9 cm (range, 2–10 cm) and 5.0 cm (range, 2–11 cm), respectively. The mean time to first flatus was 3.7 days (range, 2–7 days). The mean times to starting liquid and soft diets were 4.9 days (range, 3–22 days) and 6.6 days (range, 4–24 days), respectively. Finally, the mean postoperative hospital stay was 9.8 days (range, 6–42 days). The mean proximal margin distance of TLDG was longer than that of LADG (5.1 ± 1.4 cm vs. 4.6 ± 1.2, *P* < 0.01). There were no significant differences in other operative findings and postoperative recovery between the LADG group and the TLDG group (*P* > 0.05), although the mean operation time, blood loss and time to first flatus were slightly lower in the TLDG group than in the LADG group.

**Table 2:** Operative findings and postoperative clinical course

**Variables**	**Total (n = 240)**	**LADG (n = 93)**	**TLDG (n = 147)**	***P *** **value**
Reconstruction (Billroth I/Billroth II)	18/222	11/82	7/140	0.075
Operation time (min)	231.1 ± 48.1	238.0 ± 43.9	226.8 ± 50.2	0.080
Blood loss (mL)	136.3 ± 78.6	146.0 ± 70.5	130.1 ± 83.0	0.126
Number of retrieved lymph nodes	30.4 ± 8.6	29.5 ± 8.4	30.9 ± 8.8	0.228
Proximal resection margin (cm)	4.9 ± 1.5	4.6 ± 1.2	5.1 ± 1.4	0.002
Distal resection margin (cm)	5.0 ± 1.4	4.9 ± 1.6	5.1 ± 1.4	0.393
Time to first flatus (days)	3.7 ± 1.1	3.9 ± 1.2	3.6 ± 1.1	0.085
Time to starting liquid diet (days)	4.9 ± 1.8	4.9 ± 2.1	4.8 ± 1.6	0.794
Time to starting soft diet (days)	6.6 ± 2.4	6.7 ± 2.6	6.6 ± 2.2	0.958
Postoperative hospital stay (days)	9.8 ± 3.7	9.9 ± 3.1	9.8 ± 4.0	0.837
Postoperative complications	26	10	16	0.974
Anastomotic leakage	2	1	1	
Postoperative hemorrhage	3	1	2	
Abdominal abscess	5	3	2	
Pulmonary infection	5	2	3	
Delayed gastric emptying	6	2	4	
Pancreatic fistula	1	0	1	
Ileus	1	0	1	
Lymphorrhea	3	1	2	

The rate of postoperative morbidity was 10.8% (26/240 patients), and there was no perioperative mortality. Morbidity included two cases of anastomotic leakage at the gastrojejunostomy site (requiring surgical correction) and three cases of hemorrhage (two from the gastroduodenal artery and one from a branch of the splenic artery), two of which required a second operation to stop the bleeding. Other complications included abdominal abscess (n = 5), pulmonary infection (n = 5), delayed gastric emptying (n = 6), pancreatic fistula (n = 1), ileus (n = 1), and lymphorrhea (n = 3). These complications were controlled by conservative treatment. A comparison of morbidities in the LADG group and TLDG group and type of complications did not show any differences, and the incidence of these events was not substantially different between the groups (*P* > 0.05).

### Recurrence and survival

The mean and median follow-up was 39.7 and 31.5 months, respectively (range: 4–106 months). Among the 240 patients, survival data were available for 232 patients. Eight patients were lost to follow-up assessment, giving a follow-up rate of 96.7% (232/240) for evaluation. Forty patients developed tumor recurrence, 18 (45.0%) peritoneal recurrence, 11 (27.5%) distant or hematogenous recurrence, 7 (17.5%) lymphatic recurrence and 4 (10.0%) locoregional recurrence. Of these patients, 33 died of gastric cancer recurrence, and 7 patients are still alive with disease at closure date. Four other patients died due to causes other than gastric cancer.

**Figure 1:**
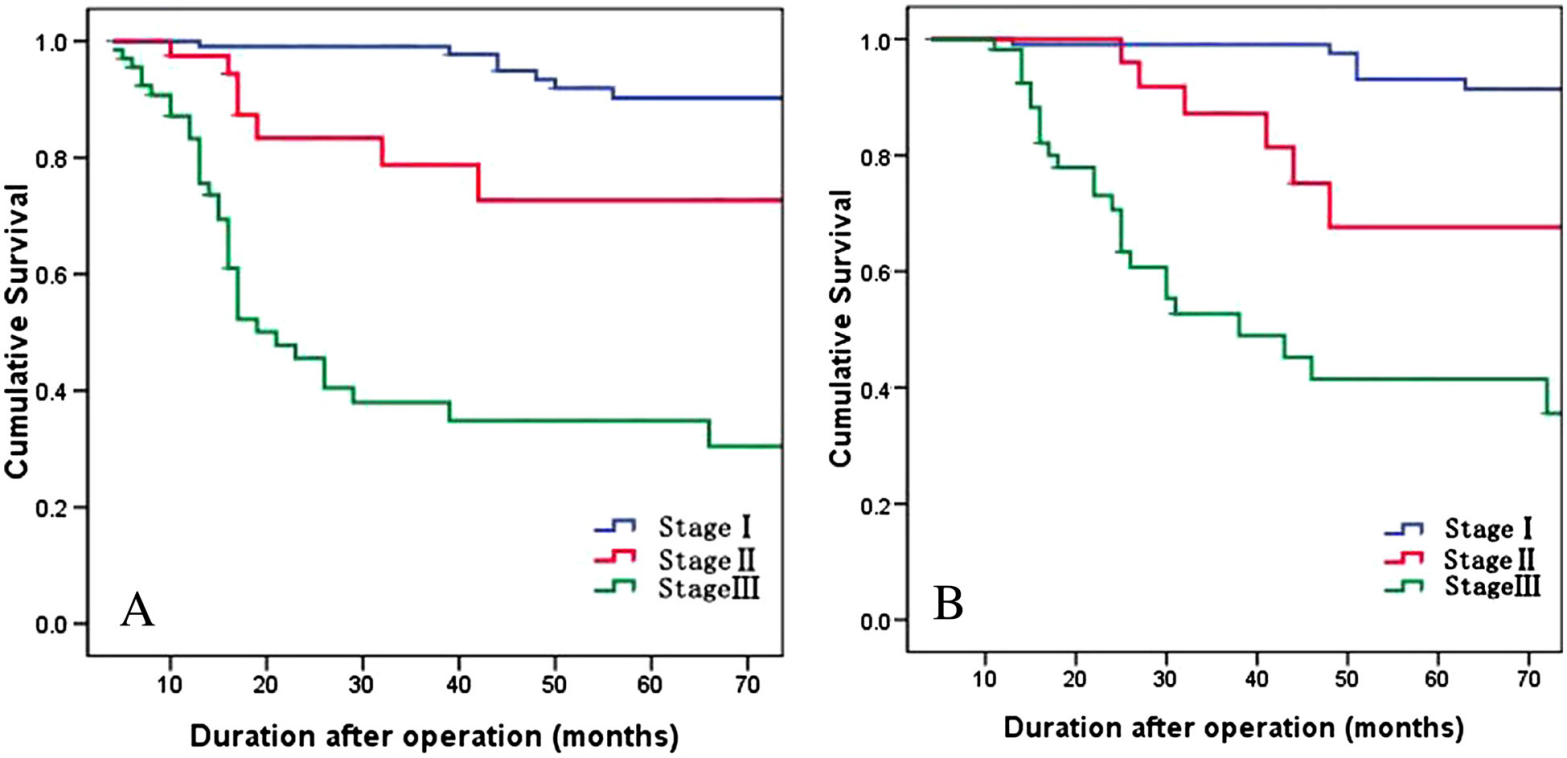
**Kaplan-Meier survival curves according to different tumor stage. (A)** Cumulative DFS. **(B)** Cumulative OS.

**Figure 2:**
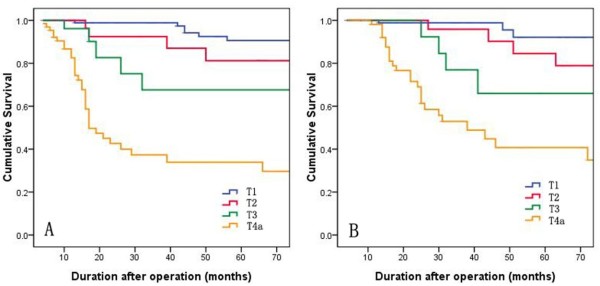
**Kaplan-Meier survival curves according to depth of invasion (T). (A)** Cumulative DFS. **(B)** Cumulative OS.

The 5-year DFS and OS in the entire cohort were 72.3% and 75.9%, respectively. According to tumor stage, 5-year DFS and OS were 90.3% and 93.1% in stage I, 72.7% and 67.6% in stage II, and 34.8% and 41.5% in stage III, respectively (Figure 1). With regard to the depth of tumor, 5-year DFS was 90.6% in T1, 81.2% in T2, 67.6% in T3, and 33.9% in T4a, and OS was 92.1%, 84.6%, 65.9%, and 40.7%, respectively (Figure 2).

## Discussion

Due to its obvious advantages over traditional gastrectomy, the number of patients undergoing LDG is rapidly increasing, especially in eastern Asia, where gastric cancer is one of the most common malignancies. The purpose of LDG in gastric cancer patients is to meet oncologic demands and not affect survival by minimizing operative insults.

However, the use of LDG in gastric cancer has not yet met with widespread acceptance because of the lack of evidence regarding the oncological adequacy of laparoscopic procedures and the long-term results [18]. Opinions still differ as to whether it can achieve the same effect in D_2_ lymphadenectomy as open surgery and whether the procedure is safe. It is well known that the adequacy of radical resection should be evaluated by the extent of lymph node dissection performed as well as the number of retrieved lymph nodes (RLNs). Laparoscopic D_2_ lymphadenectomy is a complex operation and requires abundant technical expertise due to the complicated vessels, numerous anatomical layers and the complex lymph node metastasis pathway around the stomach. Therefore, many scholars still doubt whether LDG can achieve the same radical effect as open surgery. However, some publications have already described the number of RLNs in LDG with D_2_ dissection as similar to that in open gastrectomy [19,20]. In addition, some researchers have reported not only a similar number of overall RLNs between laparoscopy and laparotomy, but also a similar number of specific lymph nodes, such as group 7, 8a, 9, 11p, 12a and 14v, which were considered difficult in laparoscopic dissection [21,22]. In our study, the mean number of RLNs per patient was 30.4 ± 8.6, which was enough for curability and to determine lymph node metastasis. Park et al. [23] evaluated the long-term results of 239 patients who underwent LDG for the treatment of advanced gastric cancer (AGC). They found that the major recurrence was distant metastasis, whereas lymph node relapses were most frequent in para-aortic or distant lymph node metastasis. Therefore, they believe that the dissection of lymph nodes around the stomach can be performed efficiently by LDG. These studies and our data suggest that oncologically appropriate D_2_ lymphadenectomy can be carried out using laparoscopic surgery.

Cancer recurrence and long-term survival rate are two critical outcomes for evaluating surgical interventions in oncological therapy. In this study, the 5-year DFS rate and OS rate after LDG were 72.3% and 75.9%, respectively, which was similar to that in previous studies [24]. In addition, the survival results stratified according to staging in the present study were comparable to historical data [25]. With regard to recurrence pattern, peritoneal recurrences were more common in our study. However, some studies demonstrated that the hematogenous pattern was most common [26,27]. Hao et al. [28] compared cancer cells following exfoliating peritoneal washing between laparoscopic and open gastrectomy for serosa-invaded AGC. The positive rates of free cancer cells were 39.68% and 44.26% in the laparoscopic and open groups, respectively, which was not significantly different. Therefore, we believe that the difference in recurrence pattern may due to the high proportion of T4a tumors in our cohort. Port-site metastasis caused by intraoperative pneumoperitoneum is another controversial issue. Shoup et al. [29] reported the long-term survival outcomes of 449 gastric cancer cases who received diagnostic laparoscopy, and only three cases developed port-site metastasis. They concluded that port-site implantation after laparoscopy was uncommon, and was not different from open incision site recurrence. This type of metastases was not reported in our study, similar to most other studies, thus we believe that pneumoperitoneum does not contribute to a higher risk of port-site metastasis.

Several previous studies [11,12,30,31] reported some advantages of TLDG over LADG, such as reduced blood loss and faster recovery. However, in our study, we could not confirm the superiority of TLDG in postoperative recovery. Because LADG is performed using a minilaparotomy in the upper abdomen, it requires a longer incision than TLDG. Therefore, some researchers argued that LADG may be associated with greater analgesic use and more pain than TLDG. However, Kim et al. [13] and our data demonstrated that the small incision used for LADG does not appear to be associated with more pain, a greater inflammatory response, or delayed recovery. In addition, the proximal margin in LADG was shorter than that in TLDG in our study. These results may be related to the nature of LADG as it is difficult to pull the proximal stomach using a narrow incision, which may influence the distance of the proximal margin. It is noteworthy that our data do not reflect the effect of TLDG in patients with a high BMI. Some researchers [32] reported that the frequency of painkiller usage was higher in obese patients who underwent LADG. This means that TLDG may be more favorable than LADG in obese patients. In our practice, however, we found that TLDG does have some advantages during intraoperative manipulation. First, TLDG is an in situ operation that avoids excessive pulling on the internal organs. When conducting LADG, the gastric stump should be pulled out of the body. This pulling places tremendous stress on the gastric stump and may even lead to tearing of the spleen envelope, causing bleeding. Also, the short gastric blood vessels must be divided, especially in patients with tumors in a high location. Conversely, intracorporeal anastomosis could reduce stress on the gastric stump and retain its blood supply and function. Second, TLDG is more suitable for a “no touch tumor” operation. When conducting LADG, the operator is limited to working through a small incision, which leads to inevitable squeezing of the tumor. There is a higher possibility that the tumor will come into direct contact with the incision. When conducting TLDG, the surgeon can achieve a “zero extrusion”. Finally, in overweight patients, the auxiliary incision of LADG may need to be extended to 8–10 cm. However, when conducting TLDG, the surgeon can simply expand the incision for the 10-mm trocar below the umbilicus to a 3–4 cm semicircle incision around the navel to enable the sample to be removed as the hypogastrium wall has more ductility.

## Conclusions

The current study demonstrated that LDG accompanied by D_2_ lymphadenectomy for gastric cancer provided an acceptable prognosis and the number of retrieved lymph nodes was considered to be oncologically acceptable. Both LADG and TLDG can be performed safely and their short-term surgical outcomes were similar. However, the results mentioned above require verification by strictly designed, large-sample, multicenter, prospective randomized studies.

## Abbreviations

LDG: Laparoscopic distal gastrectomy; LADG: Laparoscopy-assisted distal gastrectomy; TLDG: Totally laparoscopic distal gastrectomy; TNM: Tumor-node-metastasis; SD: Standard deviations; DFS: Disease-free survival; OS: Overall survival; BMI: Body mass index; RLNs: Retrieved lymph nodes.

## Competing interests

The authors declare that they have no competing interests.

## Authors’ contributions

CK and CJQ conceived and designed the study; MYP, XXW and CK performed the operations; PY, WD and ZRC collected data; CK wrote the manuscript; and MYP revised the manuscript. All authors read and approved the final manuscript.

## Pre-publication history

The pre-publication history for this paper can be accessed here:

http://www.biomedcentral.com/1471-230X/14/41/prepub
